# A new plan quality index for dose painting radiotherapy

**DOI:** 10.1120/jacmp.v15i4.4941

**Published:** 2014-07-08

**Authors:** Yang‐Kyun Park, Soyeon Park, Hong‐Gyun Wu, Siyong Kim

**Affiliations:** ^1^ Department of Radiation Oncology Massachusetts General Hospital Boston MA USA; ^2^ Interdisciplinary Program in Radiation Applied Life Science Seoul National University Seoul Korea; ^3^ Department of Radiation Oncology Seoul National University Seoul Korea; ^4^ Department of Radiation Oncology Virginia Commonwealth University Richmond VA USA

**Keywords:** dose painting, plan quality index, plan achievement, homogeneity index

## Abstract

Dose painting radiotherapy is considered a promising radiotherapy technology that enables more targeted dose delivery to tumor rich regions while saving critical normal tissues. Obviously, dose painting planning would be more complicated and hard to be evaluated with current plan quality index systems that were developed under the paradigm of uniform dose prescription. In this study, we introduce a new plan quality index, named “index of achievement (IOA)” that assesses how close the planned dose distribution is to the prescribed one in a dose painting radiotherapy plan. By using voxel‐based comparison between planned and prescribed dose distributions in its formulation, the index allows for a single‐value evaluation regardless of the number of prescribed dose levels, which cannot be achieved with the conventional indices such as conventional homogeneity index. Benchmark calculations using patient data demonstrated feasibility of the index not only for contour‐based dose painting plans, but also for dose painting by numbers plans. Also, it was shown that there is strong correlation between the new index and conventional indices, which indicates a potential of the new index as an alternative to conventional ones in general radiotherapy plan evaluation.

PACS number: 87.55.D‐

## I. INTRODUCTION

Since its introduction in 2000, dose painting (DP) in radiotherapy has been accepted as a promising technique for the treatment of cancers where high‐risk tumor subvolumes are associated within.^(1)^ The main concept of DP is prescribing and delivering spatially nonuniform doses to the target regions defined by molecular or functional imaging.^(2)^ To implement DP in clinics, two strategies about how to prescribe dose have been introduced:^(3)^ 1) contour‐based DP and 2) voxel‐based DP, which is specifically known as dose painting by numbers (DPBN). Contour‐based DP, obviously simpler to be implemented than voxel‐based DP, was already found to be effective in several clinical studies.^1^, ^4^, ^5^ On the other hand, DPBN has been an active subject of research in recent years, and its potential benefits have currently been reported by researchers.^6^, ^7^ Even though the importance of DP is increasingly recognized in radiation therapy, little attention has been paid, so far, in the aspect of plan evaluation.^(8)^ For example, single‐valued indices, such as the conformity index (CI) and the homogeneity index (HI), that are commonly used in routine clinical practice for plan evaluation need to be modified for DP planning, since such indices are formulated based on the paradigm of uniform dose prescription.

Homogeneity index (HI) (or uniformity index) is a tool to assess the planned dose distribution in a target volume. Despite its lack of detailed information compared to dose‐volume histogram (DVH), its simplicity has made it an attractive measure for quantifying the level of dose uniformity in a tumor volume. Several indexing formulas have been introduced in literatures.^9^, ^10^, ^11^ The basic concept of these methods is to calculate the ratio of the dose value of high‐dose region to the reference dose value (such as prescription dose or dose value of normal‐dose region) within a target. In DP planning, however, one cannot calculate the HI as a single index value because one plan could have multiple (in the case of contour‐based DP) or countless (in the case of DPBN) reference dose values. Moreover, since DP is basically pursuing “nonuniform” dose distribution in a target volume, assessing dose homogeneity itself may be no longer meaningful. Similarly, dose standard deviation (STD) in a target volume, which is an alternative to HI,^(12)^ is also inappropriate for the use in the evaluation of DP treatment plans.

In this study, we propose a new, simple index to score the dose distributions of DP treatment plans. The proposed index of achievement (IOA) is formulated as the volume‐weighted average of the deviation between prescription dose and planned dose. Two additional indices, index of hotness (IOH) and index of coldness (IOC), are also defined to indicate how hot or cold the target is. To evaluate the feasibility of the proposed indices, benchmark calculations were performed on sample treatment plans.

## II. MATERIALS AND METHODS

### A. Formulas of new indices

To indicate the level of achievement of a prescription goal, IOA is defined as follows:

(1) $$IOA=1+\sqrt{\sum_{i}\left[{\left(\frac{D_{i,Plan}-D_{i,Rx}}{D_{i,Rx}}\right)}^{2}\times \frac{v_{i}}{V}\right]}$$

where *V* is the total volume of the target, $v_{i}$ is the volume of the *i*th voxel in the target volume, $D_{i,Rx}$ and $D_{i,Plan}$ are the prescribed and planned dose of the *i*th voxel, respectively. We also introduce two more indices, IOH and IOC, to provide the information on the direction of deviation. The formulas of IOH and IOC are quite similar to that of IOA, except that they involve the selection of voxels where planned dose is either higher or lower than the prescribed dose as described below:

(2) $$IOH=1+\sqrt{\sum_{i}\left[{\left(\frac{D_{i,Plan}-D_{i,Rx}}{D_{i,Rx}}\right)}^{2}\times \frac{v_{i}}{V}\right]},\;(v_{i}=0\;if\;D_{i,Plan}\leq D_{i,Rx})$$

(3) $$IOC=1-\sqrt{\sum_{i}\left[{\left(\frac{D_{i,Plan}-D_{i,Rx}}{D_{i,Rx}}\right)}^{2}\times \frac{v_{i}}{V}\right]},\;(v_{i}=0\;if\;D_{i,Plan}\leq D_{i,Rx})$$

For all indices, 1 means perfect agreement between prescribed and planned dose, while value farther from 1 indicates greater dissimilarity. It should be noted that IOC has a value equal to or less than 1, representing underdose to the target volume, while IOA and IOH have values equal to or greater than 1.

### B. Feasibility evaluation in contour‐based DP cases

A well‐established example of contour‐based DP in present practice is simultaneous integrated boost intensity‐modulated radiation therapy (SIB‐IMRT).^(10)^ SIB‐IMRT delivers different dose levels to different target volumes within a single treatment fraction, allowing for both dose escalation to the tumor and better sparing of normal organs at risk (OARs).^13^, ^14^ Nasopharyngeal cancer patients (stage I or II), for instance, are often treated with SIB‐IMRT or SIB volumetric‐modulated arc therapy (SIB‐VMAT) in the institution of one of authors. To evaluate the feasibility of IOA, test calculations were performed on nine SIB‐IMRT/VMAT plans for six randomly selected nasopharyngeal cancer patients of our institution. The prescription protocol used in the selected plans is briefly described as follows:

1. PTV1 includes GTV with both primary nasopharyngeal carcinoma and gross lymph nodes; total dose is 67.5 Gy in 30 fractions.
2. PTV2 is defined as nasopharynx, posterior one‐third of nasal cavity, both parapharyngeal spaces, both pterygopalatine fossa, and high‐risk lymph node regions; prescription dose is 54 Gy in 30 fractions.
3. PTV3 is defined as low‐risk lymph nodes; prescription dose is 48 Gy in 30 fractions.

#### B.1 Calculation method using differential DVH

Differential dose‐volume histograms (dDVH), obtained from a commercial treatment planning system (Eclipse; Varian Medical Systems, Palo Alto, CA) were used in the calculation of IOA for the SIB‐IMRT/VMAT cases. Since contour‐based DP involves a few PTVs each of which has a single value of prescription dose, Eq. (1) can be modified as follows:

(4) $$IOA=1+\sqrt{\sum_{k=1}^{K}\sum_{i\in k}\left[{\left(\frac{D_{i,Plan}-D_{k,Rx}}{D_{k,Rx}}\right)}^{2}\times \frac{v_{i}}{V_{PTV(k)}}\right]}$$

where *K* is the total number of PTVs, $D_{k,Rx}$ is the prescription dose for the *k*th PTV, and $V_{PTV(k)}$ is the volume of the *k*th PTV. By binning the dose values inside the target volume, Eq. (4) can be rewritten according to the definition of dDVH as follows:

(5) $$IOA=1+\sqrt{\sum_{k=1}^{K}\sum_{j=1}^{J}\left[{\left(\frac{D_{j}-D_{k,Rx}}{D_{k,Rx}}\right)}^{2}\times \frac{dDVH_{PTV(k)}(D_{j})}{V_{PTV(k)}}\right]}$$

where *J* is the total number of bins, $D_{j}$ is the *j*th bin dose value, and $dDVH_{PTV(k)}(D_{j})$ is the absolute volume (cc) of the *j*th dose bin in the *k*th PTV. This modification formula can also be applied to the calculation of both IOH and IOC for the contour‐based DP plans.

#### B.2 Relationship between new and conventional indices

In the case of contour‐based DP, conventional indices relevant to dose homogeneity also can be obtained if calculated separately for each PTV. A comparative study was conducted to investigate the relationships between the newly introduced indices and the conventional ones. Three types of HI and STD were calculated for the same SIB‐IMRT/VMAT cases. The definition of each index is described as follows:

(6) $$HI1=\frac{D_{2}-D_{98}}{D_{Rx}}\times 100(\%)\left(10\right)$$

(7) $$HI2=\frac{D_{\max}}{D_{R}}(9)$$

(8) $$HI3=\frac{D_{5}}{D_{Rx}}(11)$$

(9) $$STD=\sqrt{\sum_{i}{\left(D_{i}-D_{mean}\right)}^{2}\times \frac{v_{i}}{V}}(12)$$

where $D_{n}$ is the corresponding minimum dose delivered to the hottest n% of the PTV, $D_{Rx}$ is the prescription dose, $D_{max}$ is the maximum dose, and $D_{mean}$ is the mean dose in the PTV.

In addition to the conventional homogeneity indices, some DVH parameters were also calculated and their correlations to the new indices were investigated. To represent ‘coldness' of the tumor (lack of coverage), 100‐V95 was chosen since V95 (i.e., PTV volume receiving 95% of prescription dose or more) has been widely used as a metric for coverage.^15^, ^16^ Similarly, V110 was calculated for representing the ‘hotness', as used for measuring dose homogeneity in previous studies.^15^, ^17^ Lastly, V95‐V110 was calculated to provide a composite metric accounting for both hotness and coldness of the tumor volume.

Since the conventional indices should be calculated for each PTV separately, the average value over three PTVs was used in the comparison. The analysis consists of two steps: 1) rating of the plan quality based on each index, and 2) evaluating correlation among these ranks by using Spearman's rank correlation test that has been shown useful in radiotherapy plan rating studies.^18^, ^19^

### C. Feasibility evaluation in DPBN cases

**Figure 1:**
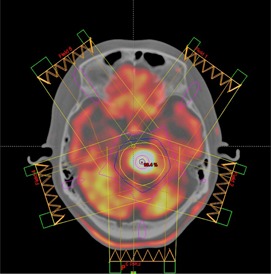
IMRT plans were made in regard to inhomogeneous prescription dose distribution based on SUV values of FDG‐PET images.

To evaluate the feasibility of the new indices for DPBN, simulated calculations were performed on two sets of fludeoxyglucose (FDG) PET‐CT images of brain cancer patients. A heterogeneous prescription dose matrix was artificially generated by linear mapping from standardized uptake value (SUV) of the PET image, with the maximum SUV matching the prescription dose of 70 Gy. Outer tumor border was defined by the 60% of the maximum SUV value. Since no inverse planning technique for DPBN is available in our commercial treatment planning system, we employed IMRT optimization in conjunction with several shell‐shaped virtual volumes having different prescription dose values to realize an inhomogeneous dose distribution within the tumor volume, as illustrated in Fig. 1. In addition to the IMRT plans, 3D conformal radiotherapy (3D CRT) plans were also made and investigated using the new indices for the comparison purpose only. In the 3D CRT planning, beam apertures were fit to the SUV‐defined tumor boundary and a prescription dose point was set on the maximum SUV point.

To calculate the new indices for DPBN, an in‐house analysis program was written in MATLAB (The MathWorks, Inc., Natick, MA). The main features of the developed software are:

1. extraction of the prescribed and planned dose matrix from the PET images and the DICOM‐RT dose files,
2. resampling to match spatial resolution between prescribed and planned dose distributions,
3. converting SUV values within a tumor volume to a prescription dose‐matrix, and
4. calculating IOA, IOH, and IOC, according to Eqs. (1) to (3).

It should be noted that, in these DPBN cases, no other contour but the tumor's outer boundary defined by the 60% of the maximum SUV value was used in the calculations.

## III. RESULTS

### A. Feasibility of using the new indices for contour‐based DP

**Figure 2:**
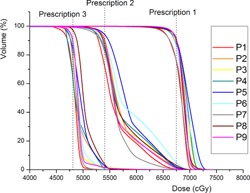
Dose‐volume histograms from nine IMRT/VMAT plans of nasopharyngeal cancer patients who were treated with simultaneous in‐field boost (SIB) technique. Three different dose prescription values (6750, 5400, and 4800 cGy) were used in each plan. Obviously, it is hard to evaluate overall plan quality with conventional indices.

Figure 2 shows DVHs of nine SIB‐IMRT/VMAT plans used for the feasibility test of the new indices. As can be seen in the figure, DVHs of PTVs under different prescriptions are significantly different from each other; thus, it would be hard to evaluate overall plan quality with conventional methods which could only calculate indices for each PTV separately. On the other hand, a single value of IOA could provide with a metric representing plan achievement over the entire target.

Table 1 summarizes the calculation result of the new indices, as well as the result of the conventional ones for the nine SIB plans. All the indices were categorized into three groups (achievement, hotness, and coldness) to be further analyzed separately. As indicated, the calculated value of the conventional index is a mean value for all three PTVs in each plan, which can be applied to only contour‐based DP cases, not DPBN cases.

**Figure 3:**
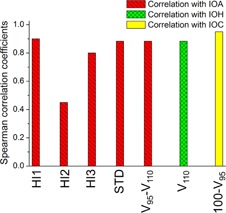
Spearman's rank correlation coefficients calculated between plan rankings from the new indices and those from the conventional ones. The calculation was carried out for the three subgroups separately: achievement, hotness, and coldness. A coefficient close to 1 indicates high degree of correlation.

Using the calculated values of the indices, individual plan rankings were made for each index. Figure 3 shows the degree of correlation between the rankings derived from the new indices and those from the conventional ones. IOA‐based ranking was found to be highly correlated with most quantities — the conventional homogeneity indices, STD and V95‐V110 indices (showing the coefficient over 0.8), except HI2 index (the coefficient of 0.45). Similarly, IOH and IOC demonstrated good correlations with V110 and 100‐V95, resulting in the coefficients of 0.88 and 0.95, respectively.

**Table 1:** The calculation result of the new and conventional indices for nine IMRT/VMAT plans

	*Achievement*						*Hotness*		*Coldness*	
*Plan No*.	*IOA*	*HI1* ^a^ *(%)*	*HI2* ^a^	*HI3* ^a^	*STD* ^a^ *(cGy)*	$V_{95}‐V_{110}$ ^a^ *(%)*	*IOH*	$V_{110}$ ^a^ *(%)*	*IOC*	$100‐V_{95}$ ^a^ *(%)*
1	1.068	15.0	1.159	1.084	213.4	91.4	1.065	7.1	0.980	1.5
2	1.070	16.9	1.185	1.123	229.7	88.9	1.069	10.5	0.990	0.5
3	1.077	17.2	1.260	1.114	236.4	90.0	1.077	9.5	0.990	0.5
4	1.086	18.0	1.179	1.126	266.3	86.6	1.083	12.5	0.977	0.9
5	1.090	18.3	1.202	1.134	247.7	85.4	1.090	14.1	0.989	0.5
6	1.092	17.7	1.187	1.128	255.5	85.2	1.091	14.2	0.988	0.6
7	1.057	15.2	1.155	1.074	189.0	94.3	1.053	3.9	0.980	1.8
8	1.076	17.5	1.202	1.131	242.7	86.0	1.075	13.4	0.988	0.6
9	1.071	16.2	1.253	1.099	212.1	90.5	1.069	8.8	0.987	0.7
Rank Criterion	C	L	C	C	L	H	C	L	C	L

a Mean value for all three PTVs.

C=closer to one is better; L=lower is better; H=higher is better.

### B. Feasibility of using the new indices for DPBN

**Figure 4:**
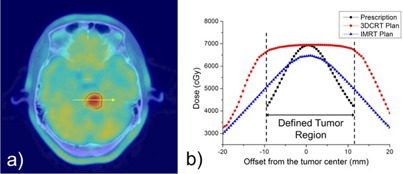
An example showing the overlay of a PET‐CT image and the tumor contour defined by the 60% of the maximum SUV (a), and the overlay profiles of the prescription and planned dose distributions from 3D CRT and IMRT plans (b).

Figure 4 shows an example result of the DPBN plans where an inhomogeneous distribution of prescription dose within the tumor volume was required. As shown in Fig. 4(b), the IMRT plan provided a nonuniform dose distribution meeting (not optimally, but at least in certain degree) the goal of DPBN plan, but the 3D CRT plan showed a uniform dose distribution across the tumor volume as is usual in the conventional dose prescription paradigm. In detail, as illustrated in Fig. 4(b), each 3D CRT plan was normalized to the maximum value of the prescription dose (7000 cGy), thus showing consistently higher doses compared to the prescription doses with no cold spots.

The new indices were successfully calculated for the DPBN cases, as summarized in Table 2. The 3D CRT plans resulted in relatively high values of IOA and IOH (approximately 1.4), indicating poor achievement and overall overdose, which was consistent with the observation described above. On the other hand, the index values of the IMRT plans were more reasonable (ranging 1.15‐1.16 for IOA, 1.14‐1.16 for IOH, and 0.96‐0.98 for IOC), obviously because of the less discrepancies between the planned and prescribed doses, confirming the feasibility of proposed indices for DPBN paradigm. Further discussions on treatment planning issues in DPBN are presented in the Discussion section.

**Table 2:** Calculation results of the new indices for two DPBN cases planned with 3D CRT and IMRT techniques

*Case No*.	*Planning Technique*	*IOA*	*IOH*	*IOC*
1	3D CRT	1.397	1.397	1.000
	IMRT	1.162	1.160	0.979
2	3D CRT	1.382	1.382	1.000
	IMRT	1.151	1.144	0.956

## IV. DISCUSSION

Biologically related models, such as tumor control probability (TCP) and normal tissue complication probability (NTCP), have been widely used for evaluating the quality of RT plans.^20^, ^21^, ^22^ In particular, several studies addressed TCP models for an inhomogeneously irradiated tumor.^23^, ^24^ In this study, we simply focused on comparing planned and prescribed physical dose distributions to indicate “achievement” in DP plans, mainly because 1) IOA is designed to be an alternative to the conventional homogeneity index that is routinely used without considering the biological effect, and 2) due to the uncertainty issue,^25^, ^26^ IOA may not necessarily be correlated with the biological effect. However, as long as clear biological data are available, we believe, proposed indices can be easily modified to incorporate such effect.

Single index method is simple and convenient, so is being commonly used in radiotherapy plan evaluation and screening. However, it is obvious that it can suffer from lack of detailed information depending on the situation. Therefore, it should be noted that the developed indices are not to replace the standard tools, such as isodose lines and DVH curves, but to provide additional information. On the other hand, as a DP‐specific alternative to the standard DVH, delta‐volume histogram (ΔVH) was recently introduced by Witte et al.^(8)^ Even though they mainly addressed cumulative ΔVH only in their study, interestingly, we found the concept of IOA could be supported by differential ΔVH(dΔVH) with the following correlation:

(10) $$IOA=1+10^{-2}\sqrt{\sum_{j=1}^{J}\left[x_{j}^{2}\times \frac{d\Delta VH(x_{j})}{V}\right]}$$

where $x_{j}$ is the jth bin of dose difference in percentage $x_{j}\%=\frac{D_{j,Plan}-D_{j,Rx}}{D_{j,Rx}}\times 100),j$ is the total number of bins, *V* is the total tumor volume, and $d\Delta VH(x_{j})$ is the differential ΔVH (in cc) corresponding to $x_{j}$.

**Figure 5:**
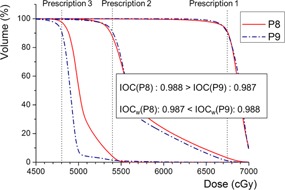
Comparison of dose‐volume histograms from the two SIB patients (patient 8 and 9 in the previous analysis). Index‐of‐coldness (IOC) was practically adjusted to IOCw by applying a weighting factor that accounts for the higher priority of the high‐dose prescription target (PTV1).

It was assumed, in this study, that each target voxel has equal amount of impact on the calculation of IOA, IOH, and IOC metrics. In principle, however, the impact of each voxel can be different from voxel to voxel. For instance, cold spots in a PTV with a higher prescription dose may be clinically more risky than those in a lower dose target. This issue may be resolved by adopting voxel‐specific weighting factors, and a simple approach is to form the weighting factor based on the relative prescription dose of each voxel. For example, Eq. (3) for IOC can be modified using the custom weighting factor as follows:

(11) $$\begin{array}{l} IOC_{w}=1-\sqrt{\sum_{i}\left[{\left(\frac{D_{i,Plan}-D_{i,Rx}}{D_{i,Rx}}\right)}^{2}\times \frac{v_{i}}{V}\times W_{i}\right],}\\ \left(\cdot v_{i}=0\;if\;D_{i,Plan}\geq D_{i,Rx}\;\cdot W_{i}={\left(\frac{D_{i,Rx}}{D_{mean,Rx}}\right)}^{2}\right) \end{array}$$

where $W_{i}$ is the sample weighting factor, defined as the square of the ratio of the voxel‐prescription dose and the mean prescription dose $\left(D_{mean,Rx}\right)$ over the entire target volume. Figure 5 demonstrates the effect of the weighting factor by comparing the DVHs of the two SIB patients (Patients 8 and 9 from Fig. 2). It was shown that the IOC‐based ratings can be reversed when the weighting factor applied. A more accurate weighting factor system can be established when biological importance of hotness and coldness becomes much clearer in the future.

Another limitation of this study is that the proposed indices are applicable to target volumes only and not to normal/critical organs. This limitation can be justified, at present, by the fact that the DP technique was originally designed for target volumes where relevant molecular/functional imaging information is available.^(2)^ In theory, however, the DP technique can also be specifically used for normal/critical structure/region sparing, as inferred from the previous studies.^27^, ^28^ We expect that other indices specific for normal tissue sparing will be proposed if the spatially‐varying dose constraint in a normal organ (dose‐constraint painting) is available in the future.

In the benchmark calculations of the DPBN cases, it was found that the values of the indices are relatively farther from the ideal value compared to those of the contour‐based DP cases. It is mainly because the planned dose distributions of DPBN were not fully optimized due to the incapability of the RTPS to perform voxel‐based optimization. However, we believe this limitation has little impact and would not alter the findings of this study.

## V. CONCLUSIONS

We have proposed a new plan quality index to assess how close the planned dose distribution is to the prescribed one for DP radiotherapy. In contour‐based DP cases, the applicability of the introduced index was demonstrated by showing strong correlations with diverse conventional homogeneity and DVH indices. It was also shown that the new index is feasible to be used in DPBN cases where conventional indices cannot be applied due to the varying dose prescription levels within the tumor volume. We believe that our study is a good first step towards establishing a new paradigm of plan quality indexing compatible with DP radiotherapy.

## ACKNOWLEDGMENTS

The authors appreciate the effort of Jangpil Park, Dosimetrist of Seoul National University Hospital, for assisting with IMRT/VMAT treatment plans used in this study.
